# Review: Adaptation of Beneficial Propionibacteria, Lactobacilli, and Bifidobacteria Improves Tolerance Toward Technological and Digestive Stresses

**DOI:** 10.3389/fmicb.2019.00841

**Published:** 2019-04-24

**Authors:** Floriane Gaucher, Sylvie Bonnassie, Houem Rabah, Pierre Marchand, Philippe Blanc, Romain Jeantet, Gwénaël Jan

**Affiliations:** ^1^STLO, Agrocampus Ouest, Institut National de la Recherche Agronomique, Paris, France; ^2^Bioprox, Levallois-Perret, France; ^3^Science de la Vie et de la Terre, Université de Rennes 1, Rennes, France; ^4^Pôle Agronomique Ouest, Bba, Rennes, France

**Keywords:** stress, probiotic, adaptation, drying, osmo regulation

## Abstract

This review deals with beneficial bacteria, with a focus on lactobacilli, propionibacteria, and bifidobacteria. As being recognized as beneficial bacteria, they are consumed as probiotics in various food products. Some may also be used as starters in food fermentation. In either case, these bacteria may be exposed to various environmental stresses during industrial production steps, including drying and storage, and during the digestion process. In accordance with their adaptation to harsh environmental conditions, they possess adaptation mechanisms, which can be induced by pretreatments. Adaptive mechanisms include accumulation of compatible solutes and of energy storage compounds, which can be largely modulated by the culture conditions. They also include the regulation of energy production pathways, as well as the modulation of the cell envelop, i.e., membrane, cell wall, surface layers, and exopolysaccharides. They finally lead to the overexpression of molecular chaperones and of stress-responsive proteases. Triggering these adaptive mechanisms can improve the resistance of beneficial bacteria toward technological and digestive stresses. This opens new perspectives for the improvement of industrial processes efficiency with regard to the survival of beneficial bacteria. However, this bibliographical survey evidenced that adaptive responses are strain-dependent, so that growth and adaptation should be optimized case-by-case.

## Introduction

Bacteria may constitute useful fermentation starters, healing probiotics, or both for the so-called “2-in-1” bacteria. These beneficial bacteria, within fermented foods as starter or within functional foods supplements as probiotic are ingested in high amount and it is a means to modulate the activity of the human gut microbiota (Collins and Gibson, 1999; Parvez et al., 2006; Moens et al., 2017). The ingested bacteria are essential for normal development of the immune system (Marco et al., 2017). Indeed, gut microbiota dysbiosis is increasingly correlated to various diseases, such as inflammatory bowel diseases and obesity, which are on the rise and constitute a public health problem linked to the Western diet (David et al., 2014; Plé et al., 2016). In order to decrease the consequences of such health problems, probiotics and fermented foods could be part of the solution. Indeed, the size of the probiotic market exceeded US$42 billion in 2016 and is expected to exceed US$64 billion in 2022 (Marketsandmarkets.com, 2017). Probiotics are also increasingly incorporated into non-fermented functional foods such as infant formula, ice creams, and cereal bars (Homayouni et al., 2008; Braegger et al., 2011; Bampi et al., 2016). These products constitute a growing market as well. The starter culture market, worth US$1.0 billion by 2018, is projected to grow at a CAGR of 5.6% (Marketsandmarkets.com, 2014).

Starters and probiotics are usually dried to produce easy-to-use ingredients that are stable and flexible for different applications like food, feed, and pharmaceutical products. At the industrial scale, two types of drying processes are implemented: freeze-drying and spray-drying. In addition, drying is a way to keep bacteria alive on the long term at ambient temperature and to facilitate their storage and transport. At the industrial level, the use of frozen starter culture has the disadvantage of high energy costs during transportation and storage. Therefore, frozen starters go hand-in-hand with high operating costs (Santivarangkna et al., 2007). During powder production, storage, and digestion, bacteria of interest may encounter multiple stresses, which may affect its survival and its beneficial effects. Indeed, they must survive during powder production and storage in a first time, and in a second time, during fermentation (starter) and digestion (probiotics) (Picot and Lacroix, 2004). A stress is defined as “any change in the genome, proteome or environment that imposes either reduced growth or survival potential. Such changes lead to attempts by a cell to restore a pattern of metabolism that either fits it for survival or faster growth” (Booth, 2002). Different pretreatments can induce an enhanced tolerance to various stresses, which may occur during bacterial powders production and consumption. In this paper, we reviewed stresses encountered during drying processes, storage, and digestion. Then, we summarized the main molecular adaptation mechanisms induced by different pretreatments described in propionibacteria, lactobacilli, and bifidobacteria, recognized as beneficial bacteria. We then describe the impact of the adaptation mechanisms induced by pretreatments on tolerance toward technological and digestive stresses. These data should contribute to making an informed choice of the best treatments for reinforcing bacteria during drying, storage, and digestion.

## Beneficial Effects of Propionibacteria, Bifidobacteria and Lactobacilli

### General Features of Propionibacteria, Bifidobacteria, and Lactobacilli

This review is focused in propionibacteria, lactobacilli, and bifidobacteria, which are generally recognized as safe (GRAS) (Avalljaaskelainen and Palva, 2005; Picard et al., 2005; Cousin et al., 2010). They constitute, to the best of our knowledge, the main bacterial genera considered as starter or/ and as probiotics.

Propionibacteria and bifidobacteria both belong to the Actinobacteria class, which comprises non-sporulating Gram-positive bacteria with a high G+C content (Cousin et al., 2010). Propionibacteria are currently described as non-motile pleomorphic rods. Their optimal growth temperature is 30°C with a pH of 7.0. They are anaerobic aerotolerant, and many of them are catalase-positive. Propionibacteria can use a wide range of carbon sources such as organic acids (lactate), carbohydrates (lactose, glucose, galactose, and fructose), and alcohol (glycerol) (Cousin et al., 2010). They are hetero-fermentative bacteria and their favorite substrate is lactate. For a consumption of 3 moles of lactate, propionibacteria produce 1 mole of acetate, 2 moles of propionate, and 1 mole of carbon dioxide, according to the Fitz equation (Gagnaire et al., 2015). Bifidobacteria require cysteine in their growth medium, consume carbohydrates, and produce lactate and acetate at molar ratio of 2:3, respectively, thus decreasing the pH of the culture medium. Their optimal growth temperature is 37°C (Hsu et al., 2007). Bifidobacteria are bifid or multiple-branched rods, and are strictly anaerobic and catalase-negative. They are naturally present in the gastrointestinal tract and vagina of animals (Schell et al., 2002; Savijoki et al., 2005; De Dea Lindner et al., 2007).

Lactobacilli are Gram-positive, firmicute bacteria with a low G+C content. Their natural habitat includes the digestive or reproductive tract of animals, raw milk, decomposing plants, and fermented products. They are anaerobic, non-sporuling, and facultative heterofermentative. Lactobacilli require rich growth media that contains carbohydrates, amino acids, peptides, fatty acid esters, salts, nucleic acid derivatives, and vitamins (Chervaux et al., 2000; Elli et al., 2000). Lactobacilli optimal growth temperature is generally between 30 and 40°C (Hammes and Hertel, 2015). Like bifidobacteria, lactobacilli use carbohydrates (as carbon and energy source) and produce lactic acid, therefore inducing a significant decrease in the pH of the medium. Among lactic acid bacteria, members of the genera *Streptococcus*, *Lactococcus*, *Leuconostoc*, and *Pediococcus* are also widely used as starters. The majority of available literature on probiotic effects and/or studies deals with lactobacilli, the largest genus within the group of lactic acid bacteria. In addition to lactobacilli, this review also deals with propionibacteria and bifidobacteria, used as probiotics and/or as starters.

### Potential of Propionibacteria, Bifidobacteria, and Lactobacilli as Probiotics

Probiotics are defined as “live microorganisms that, when administered in adequate amounts, confer a health benefit on the host” (FAO/WHO, 2001). Potential probiotics can be isolated from many sources (Mills et al., 2011). A minimum of 10^7^ live probiotic bacterial cells per gram or milliliter of product at the time of consumption is recommended by the International Dairy Federation (IDF) (Huang et al., 2017). Since these bacteria are included in probiotic preparations, the amount of live bacteria in these latter (tablets or capsules) has to be optimized (Gagnaire et al., 2015; Huang et al., 2017), as does their viability during digestion (Kailasapathy and Chin, 2000; Rabah et al., 2018). Probiotic bacteria can enhance or preserve health, in strain-dependent manner among probiotic species (Lebeer et al., 2008; Le Maréchal et al., 2015; Plé et al., 2016).

As mentioned previously, the consumption of probiotics modulates the gut microbiota, which is a pivotal effect. Indeed, the presence of bifidobacteria in the intestine is very important, especially during the first years of life (De Dea Lindner et al., 2007), when they represent a majority of the intestinal microbiota. This part decreases with time over a lifetime (Sánchez et al., 2013).

The probiotic effects include also immunomodulation, described in propionibacteria, bifidobacteria, and lactobacilli, which prevent and help to treat various immunes diseases as inflammatory bowel diseases or allergy (Picard et al., 2005; Steed et al., 2010; DuPont et al., 2014; Rong et al., 2015; Saez-Lara et al., 2015). Indeed, this early colonization of the gut by bifidobacteria and propionibacteria seems to prevent necrotizing enterocolitis (Colliou et al., 2017; Young et al., 2017). In addition, the manipulation of the gut microbiota with probiotics has been considered as a possible manner to prevent and treat obesity and cancers (Saez-Lara et al., 2015; Dasari et al., 2017; Rabah et al., 2017; Brusaferro et al., 2018). *Propionibacterium freudenreichii* inhibits pathogens such as *Salmonella* Heidelberg (Nair FIM 2018) and meticillin-resistant *Staphylococcus aureus* (Sikorska and Smoragiewicz, 2013). *Lactobacillus amylovorus* inhibits *Escherichia coli* adhesion to intestinal cells (Hynönen et al., 2014) while *Lactobacillus plantarum* inhibits *E. coli* 0157:H7 adhesion to collagen (Yadav et al., 2013). Selected strains of bifidobacteria were reported to inhibit growth and toxicity of *Clostridium difficile* (Valdés-Varela et al., 2016), or to affect the virulence of *Listeria monocytogenes in vitro* (Rios-Covian et al., 2018). They may protect against gastrointestinal disorders (Sanchez et al., 2007) and prevent diarrhea through their effects on the immune system and/or through enhanced resistance to colonization by pathogens, as *C. difficile* (Cantero et al., 2018). Therefore, alleviation of lactose intolerance symptoms is demonstrated by lactobacilli, which provide the missing enzyme for lactose-intolerant people and, therefore, are complementary to a host that is deficient in β-galactosidase (Levri et al., 2005; Pakdaman et al., 2015). The preclinical studies are promising and data highlight the strain-dependent aspect of these effects among propionibacteria, bifidobacteria, and lactobacilli species. However, more investigations are needed to assess the probiotic effectiveness at clinical level and to determine the precise molecular mechanisms involved (Khalesi et al., 2018).

The molecular mechanisms identified in probiotics effects seem to be similar among propionibacteria, lactobacillia, and bifidobacteria species. They include the production of secreted metabolites as short fatty acids (Lan et al., 2008; LeBlanc et al., 2017; Rabah et al., 2017), and the presence of key surface components (Konstantinov et al., 2008; Foligné et al., 2013; Sengupta et al., 2013; Taverniti et al., 2013; Le Maréchal et al., 2015; Lightfoot et al., 2015; Sarkar and Mandal, 2016; Louis and Flint, 2017; do Carmo et al., 2018). In the dairy propionibacterium *P. freudenreichii*, the immunomodulatory properties are linked to the ability of selected strains to induce the release of the regulatory IL-10 by immune cells (Foligne et al., 2010). This property is mediated by surface proteins of the S-layer family (Le Maréchal et al., 2015; Deutsch et al., 2017). Accordingly, propionibacteria belonging to the human gut microbiota protect new-borns from necrotizing enterocolitis via Th17 cell regulation (Colliou et al., 2017). This property is also dependent on proteins of the surface layer and induces the generation of bacteria-specific Th17 cells, while maintaining IL-10^+^ regulatory T cells (Ge et al., 2019). In *Lactobacillus acidophilus*, immunomodulatory properties are dependent on the surface-layer protein SlpA, it occurs via binding to DC-SIGN receptors on dendritic cells and inducing a concentration-dependent production of IL-10 (Konstantinov et al., 2008). This binding plays a pivotal role in *L. acidophilus* ability to mitigate induced colitis (Lightfoot et al., 2015). By contrast, in the probiotic *Bifidobacterium bifidum*, such a protective ability, as well as the immunomodulatory properties, are linked to the presence of cell surface polysaccharides. These last also act via regulatory dendritic cells, but through a partially Toll-like receptor 2-mediated mechanism, inducing the generation of Foxp3+ regulatory T cells (Verma et al., 2018). The strain-dependent nature of the probiotics beneficial effects is closely correlated to the strain-dependent nature of the ability of probiotics to express or produce these probiotics effectors.

### Potential of Propionibacteria, Bifidobacteria, and Lactobacilli as Starters

Fermented foods have been produced and consumed worldwide for centuries (Ebner et al., 2014; Marco et al., 2017). Fermented products can be produced from dairy, meat, or plant matrices and then used to produce a large diversity of fermented foods. As a result, there is a multitude of food matrix-starter combinations. Some fermentations can be spontaneous, but many products require inoculation by starters (Rong et al., 2015; Walsh et al., 2016; Marco et al., 2017). To produce fermented foods, a large number of bacteria may be used. This number was shown to be stable over time and between countries (Fortina et al., 1998; De Dea Lindner et al., 2007), although the diversity of fermented foods tends to decrease because of industrialization (Marco et al., 2017).

Lactobacilli are extensively used as starters in the fermentation of dairy products, e.g., *L. acidophilus* in cheese, *Lactobacillus delbrueckii* in yogurt, and *Lactobacillus kefiranofaciens* in kefir. *L. acidophilus* is also used in fermented plant products such as kimchi (Meira et al., 2015; Marco et al., 2017; Wang et al., 2018), that also provides other lactobacilli such as *Lactobacillus sakei* (Kwon et al., 2018), *L. plantarum* (Lim et al., 2018), and *Lactobacillus fermentum* (Yoo et al., 2017). Propionibacteria are widely used as ripening starters in the manufacture of Swiss-type cheeses (Thierry et al., 2011) and also contribute to the fermentation of vegetable products (Yu et al., 2015). Although not really considered as fermentation starters, bifidobacteria can be added before the fermentation process, with an impact on the final organoleptic properties of the product (Kailasapathy, 2006).

During fermentation, bacteria modify the matrices and contribute to the final flavor, texture, nutrition, and organoleptic qualities (Marco et al., 2017). They offer an increased availability of bioactive molecules, vitamins, and other constituents due to the process of fermentation. Thus, fermentation leads to increased digestibility of dairy products and plant matrices (Marco et al., 2017; Moens et al., 2017). Fermented dairy products have a low lactose content. Indeed, this carbohydrate is digested by the starter (Guarner et al., 2005), like other oligosaccharides (Sánchez et al., 2013; Moens et al., 2017). Furthermore, starters improve food storage and preservation, and may thus make it possible to decrease the use of additives (Mende et al., 2016).

Traditional fermented foods products can have a probiotic effect *per se* (Levri et al., 2005). Combining selected strains of probiotic bacteria with two-in-one abilities (both efficient starters and probiotics) leads to new functional fermented foods (Dimitrellou et al., 2016). Probiotics can be added to foods like in the case of bifidobacteria in yogurt (Picot and Lacroix, 2004), but these type of processes require adequate technologies to keep bacteria alive (Racioppo et al., 2017). The use of probiotics in food is growing, but mechanisms used by bacteria to exert health benefits are not fully elucidated (Savijoki et al., 2005). The main troubles targeted are antibiotic-associated diarrhea, traveler’s diarrhea, pediatric diarrhea, inflammatory bowel disease, and irritable bowel disease. Although there is a limited number of clinical studies with fermented foods (Marco et al., 2017), preclinical studies show promising positive effects (Chen et al., 2014; Gao et al., 2015; Kato-Kataoka et al., 2016; Plé et al., 2016). All the above-described probiotic effects should be considered as potential effects. Indeed, the European Food Safety Authority (EFSA) requires substantial clinical proof before allowing a functional claim.

For all probiotics and starters, survival during industrial production processes, storage step, and supply chain is a prerequisite. For *in situ* probiotic efficacy, effects that rely on the local production of beneficial metabolites such as short-chain fatty acids or vitamins require live bacteria capable of surviving digestive tract constraints. In contrast, for effects that rely on cellular fractions such as cell wall immunomodulating compounds, viability may be less crucial. Anyway, the probiotic bacterium should adapt industrial constraints to keep alive and then used.

## Stress Encountered During Industrial Production and Digestion Processes

### Industrial Drying Process and Storage

#### Freeze-Drying Process

Frozen starter culture is benchmark, as a high cooling rate and very low temperature (-80°C) permit to increase bacteria viability. However, the use of frozen starter culture at the industrial level has the disadvantage of requiring negative temperatures during transportation and storage. Therefore, frozen starters go hand-in-hand with high operating costs (Santivarangkna et al., 2007). Freeze-drying is the most conventional process, i.e., the most frequently used with regard to its efficiency. The drying process of bacteria is conducted by sublimation, with the advantage of providing high bacterial viability (Santivarangkna et al., 2007). However, this process is discontinuous and expensive. Moreover, the ice crystals produced during freezing in the intracellular and extracellular compartments may be responsible for cell damage: compromised cellular integrity, broken DNA strands, altered transcription and replication, and reduced membrane fluidity. Freeze-drying favors also the appearance of holes in the cell membrane, which may cause cell death if another stress occurs (Carvalho et al., 2004; Giulio et al., 2005). During the freezing step, the cooling rate is an important factor for maintaining bacterial viability. In addition, bacteria suffer from osmotic stress (decrease in surrounding water activity) during freeze-drying, that represents a common constraint encountered during the drying of bacteria. The composition of drying media is highly studied in the aim of improving bacterial viability during the process. As an example, skim milk can be used as a drying matrix since it has the advantage of stabilizing the cell membrane constituents and contains proteins that build a protective coating for the bacteria (Carvalho et al., 2004). Protective agents can be added to the drying matrix in order to improve bacterial viability during storage. These molecules need to have an amino group, a secondary alcohol group, or both (Carvalho et al., 2004). Suitable protective agents should provide cryoprotection for the bacteria during freezing, be easily dried, and improve drying matrix stability and rehydration (Zhao and Zhang, 2005). Several sugars are used to protect bacteria, e.g., glucose, fructose, lactose, and trehalose. Sugar alcohols like sorbitol and inositol can also be used. Monosodium glutamate can stabilize protein structure. Antioxidants such as ascorbate can be also added in order to protect membrane lipids against damage caused by the freeze-drying process (Carvalho et al., 2004; Kurtmann et al., 2009). The efficiency of these different agents to limit freeze-drying stresses is bacteria dependent (Zhao and Zhang, 2005).

#### Spray-Drying Process

Spray-drying decreases the cost of water removal by a factor of six, in comparison to freeze-drying (Paéz et al., 2012), while producing powder with a yield four to seven times higher than that of freeze-drying (Golowczyc et al., 2011; Huang et al., 2016a). Spray-drying has the advantage of being a continuous process, drying and encapsulating bacteria occurred in the same time (Peighambardoust et al., 2011). This process constitutes thus an emerging alternative to freeze-drying (Huang et al., 2016b).

Spray-drying can be divided into two steps: an initial constant rate evaporation stage, which is at the wet-bulb temperature, and the falling rate evaporation stage, which leads to product temperature increases toward the outlet air temperature at the end of drying (Huang et al., 2017). The time–temperature combination is an important factor that determines the extent of cell death, in particular depending on the outlet temperature value (Santivarangkna et al., 2007). The spray-drying process usually leads to high temperatures over a short time. Heat stress is a commonly encountered stress, affecting bacterial viability during spray-drying. Cell death is due to the destruction of more than one critical component (Peighambardoust et al., 2011), including major damage to membrane lipids and/or aggregation of membrane protein (Mills et al., 2011). Indeed, the membrane bilayer structure is thermodynamically unstable (Peighambardoust et al., 2011) so that damage caused to the membrane is the first cause of viability losses (Simpson et al., 2005). Other damage may occur inside the cell during drying. It affects DNA (Simpson et al., 2005), intracellular proteins, and ribosomes (Mills et al., 2011). RNAs may also be impaired as a result of the escape of Mg^2+^ (Huang et al., 2017). Indeed, the use of lower temperatures during spray-drying is possible to enhance viability, but the high water activity (a_w_) obtained in the powder limits its stability during storage. Moreover, bacteria suffer from oxidative and osmotic stresses that are coupled to heat stress during spray-drying (Huang et al., 2017), first, from the drying air and, second, because of the concomitant increase in osmotic pressure with the loss of water (Desmond et al., 2001).

Survival rate during spray-drying is species and strain-dependent, which is related to species- and strain-dependent heat tolerance ability (Peighambardoust et al., 2011). *P. freudenreichii* has a better chance to survive than *Lactobacillus casei* (Huang et al., 2016a), and thermotolerant *S. thermophilus* survives better than lactobacilli (*L. delbrueckii* ssp *bulgaricus* and *L. acidophilus*) (Huang et al., 2017). One of the most important factors for spray-drying survival is the intrinsic resistance to heat that is species- and strain-dependent and determine the survival of the bacteria during the process. The difference in heat resistance is more important when the heat challenge increases (Paéz et al., 2012). The thermic intrinsic sensitivity of a strain is essential to determine survival (Simpson et al., 2005) and can be optimized to enhance viability (Santivarangkna et al., 2007). In contrast, the oxygen tolerance of bifidobacteria is not correlated with a good viability during drying (Simpson et al., 2005).

Statistical models were developed to predict survival during spray-drying based on the hypothesis that bacterial death during spray-drying followed a probability distribution (Perdana et al., 2014). However, this prediction was not very precise because bacteria can be pre-adapted to technological stress, and because the drying medium may play a protective role. Viability can be improved by adding either trehalose, which stabilizes membranes and proteins and decreases membrane phase transition temperatures, or gelatin, gum Arabic, or fruit juice (Huang et al., 2017). It is thus possible to modulate the drying media composition to enhance bacterial survival (Sollohub and Cal, 2010). However, adding protective agents to the drying medium will not necessarily lead to optimal bacteria survival during storage (Santivarangkna et al., 2007).

#### Storage

During storage, bacterial viability generally remains higher for freeze-dried powders compared to spray-dried powders (Santivarangkna et al., 2007), which may be due to heat stress (Huang et al., 2017). Viability indeed decreases during storage, particularly at ambient temperature (Simpson et al., 2005). During storage, the water activity of the dry product, the glass transition temperature, the environmental relative humidity, temperature, and light are important factors for maintaining cell viability (Santivarangkna et al., 2007; Huang et al., 2017). For freeze-dried powders, stability is greater at low temperatures and in oxygen-free environments (Santivarangkna et al., 2007). Viability is inversely correlated to temperature (Paéz et al., 2012), but industry generally looks for stable dry cultures at ambient temperature for marketing purposes. The presence of oxygen decreases viability because of lipid oxidation. Some authors therefore attempted to add antioxidants to decrease the impact of oxidative stress, but the results were not convincing for long-term storage (Santivarangkna et al., 2007).

### Digestion

Some probiotic effects require live bacteria in sufficient amounts in the large intestine. Probiotic bacteria have to pass the stomach, which seems to be the most difficult part of the gastrointestinal tract to cross during digestion. Bacteria have to survive the acid conditions and the presence of bile salts (Mainville et al., 2005). The harsh conditions of the gastrointestinal tract also include variations in the redox potential, and the presence of hydrolytic enzymes such as lysozyme, of various proteases and lytic compounds found in pancreatin, and of bile salts. Acid stress is a major limit to fermentation by beneficial bacteria. A decrease in the pH is generally the reason why fermentation stops. Probiotics such as bifidobacteria are also added to acidic yogurt-type products and then stored at 4°C before consumption. Finally, all the beneficial bacteria face severe acid stress during the first step of digestion (i.e., stomach), which limits the *in situ* activity. Viability in the intestinal compartment is specie and strain-dependent (Picot and Lacroix, 2004; Alcantara and Zuniga, 2012). For example, *L. casei* and other lactobacilli have low pH tolerance (Broadbent et al., 2010), although it is still higher than that of some bifidobacteria (except for *Bifidobacterium infantis*). The capacity to resist bile salts is strain-dependent, and probiotic strains can thus be selected for their tolerance to bile salts. Bile salts act as detergents (Alcantara and Zuniga, 2012; Papadimitriou et al., 2016) and cause cell damage and cytotoxicity (Arnold et al., 2018). Molecular mechanisms leading to bile adaptation were evidenced in dairy propionibacteria (Leverrier AEM2003, arch 2004), in lactobacilli (Ruiz FIM 2013, Goh MolCelFact 2014, Vinusha 2018), and in probiotic bifidobacteria (Ruiz FIM 2013, Sanchez AEM 2007).

Lactobacilli and bifidobacteria have bile salts hydrolase *(bsh)* genes, which can have a positive or negative impact on bile salts tolerance, so the selection requires a careful examination of microbial physiology (Arnold et al., 2018). Since osmolarity is constantly changing in the gastrointestinal tract, bacteria survival may be affected byf osmotic stress (De Dea Lindner et al., 2007).

## Mechanisms of Stress Adaptation Triggered by Pretreatements

**FIGURE 1:**
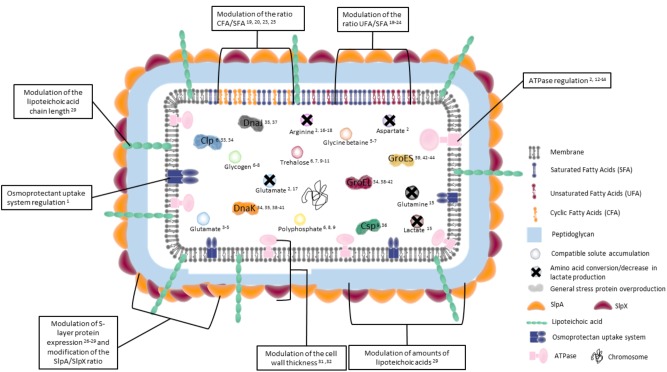
Key actors of adaptive mechanisms in bacteria during osmotic, acid, oxidative, heat, cold, and bile salts adaptation. General adaptive bacterial mechanisms during osmotic, acid, oxidative, heat, cold, and bile salts treatment are represented. Peptidoglycan is represented in blue. Membrane lipids under normal growth are represented in gray. Amounts of saturated (blue), unsaturated (red), and cyclic (yellow) fatty acids are modulated by treatments. S-layer proteins, which may be involved in adaptation, are represented in yellow and red outside the peptidoglycan. Liptechoic acids, whose length is modulated, are presented in green. Inducible transmembrane ATPase and osmoprotectant uptake systems are represented in pink and blue, respectively. In the cytoplasm, general stress proteins are represented by different colors. Colored circles represent different osmoprotectant and energy storage compounds. Crosses on circles mean the conversion of the molecule. The chromosome is represented in black. The numbers indicate corresponding references in the tables.

Beneficial bacteria used as technological starters will have to face technological stresses such as heat, cold, oxidative, and osmotic stresses. Probiotic bacteria should also survive digestive stress, in addition to the above-mentioned stresses. Digestive stresses include acid, bile salts, and osmotic constraints. The main adaptive mechanisms triggered by bacteria to deal with such damage are DNA repair, metabolic pathways of lipid modification, chaperones and proteases, accumulation of compatible solutes, and reactive oxygen specices (ROS) detoxification (Desmond et al., 2001; Sheehan et al., 2006; Li et al., 2009). The medium culture choice is crucial since its composition may induce different adaptation mechanisms, as detailed below. General adaptive mechanisms induced by different pretreatments of propionibacteria, bifidobacteria, and lactobacilli are presented in Figure 1. Adaptive mechanisms are induced by specific pretreatments reported in Table 1.

**Table 1:** Adaptive mechanisms induced by stressing conditions or by modifications of the growth medium in bifidobacteria, propionibacteria, and lactobacilli.

Adaptive mechanism	Stress	Bacteria	References	Corresponding number in the figures
ABC transporter	Heat	*L.*^a^ *rhamnosus*	Prasad et al., 2003	1^∗^
Arginine accumulation	Addition of arginine	*P.*^b^ *acidopropionici*	Guan et al., 2013	2
Glutamate accumulation	Addition of glutamate	*L. sakei*	Ferreira et al., 2005	3
Glutamate accumulation	Osmotic	*L. plantarum*	Glaasker et al., 1996; Kets et al., 1996	4, 5
Glycine betaine accumulation	Osmotic	*P. freudenreichii*	Huang et al., 2016b	6
Glycine betaine accumulation	Osmotic	*L.*^c^ *lactis*	Romeo et al., 2003	7
Glycine betaine accumulation	Osmotic	*L. plantarum*	Glaasker et al., 1996	5
Glycogen accumulation	Addition of carbon (raffinose and trehalose)	*L. acidophilus*	Goh and Klaenhammer, 2014	8
Glycogen accumulation	Cold	*P. freudenreichii*	Dalmasso et al., 2012b	7
Glycogen accumulation	Osmotic	*P. freudenreichii*	Huang et al., 2016b	6
PolyP accumulation	Addition of polyphosphate	*Lactobacillus*	Alcantara et al., 2014	8
PolyP accumulation	Cold	*P. freudenreichii*	Dalmasso et al., 2012a	9
PolyP accumulation	Osmotic	*P. freudenreichii*	Huang et al., 2016b	6
Trehalose accumulation	Acid	*P. freudenreichii*	Cardoso et al., 2007	10
Trehalose accumulation	Cold	*P. freudenreichii*	Dalmasso et al., 2012a,b	9, 7
Trehalose accumulation	Osmotic	*P. freudenreichii*	Cardoso et al., 2004; Huang et al., 2016b	11,6
Trehalose accumulation	Oxydative	*P. freudenreichii*	Cardoso et al., 2007	10
F_0_F_1_-ATPase upregulated	Acid	*B.*^d^*longum*	Sanchez et al., 2007	12
F_0_F_1_-ATPase upregulated	Acid	*L. rhamnosus*	Corcoran et al., 2005	13
F_0_F_1_-ATPase upregulated	Acid	*P. acidopropionici*	Guan et al., 2013	2
F_0_F_1_-ATPase upregulated	Addition of glucose	*L. rhamnosus*	Corcoran et al., 2005	13
F_0_F_1_-ATPase upregulated	Bile salt	*B. animalis*	Sanchez et al., 2006	14
F_0_F_1_-ATPase upregulated	Increase of NAD/NADH	*P. acidopropionici*	Guan et al., 2013	2
L-lactate deshydrogenase downregulated	Acid	*L. rhamnosus*	Koponen et al., 2012	15
Arginine conversion	Acid	*L. reuteri*	Rollan et al., 2003; Teixeira et al., 2014	16, 17
Arginine conversion	Acid	*P. acidopropionici*	Guan et al., 2013	2
Arginine conversion	Heat	*L. fermentum*	Vrancken et al., 2009	18
Arginine conversion	Osmotic	*L. fermentum*	Vrancken et al., 2009	18
Aspartate conversion	Acid	*P. acidopropionici*	Guan et al., 2013	2
Glutamate conversion	Acid	*L. reuteri*	Teixeira et al., 2014	17
				
Glutamate conversion	Acid	*P. acidopropionici*	Guan et al., 2013	2
Glutamine conversion	Acid	*L. reuteri*	Koponen et al., 2012	15
Decrease in unsaturated/saturated fatty acid ratio	Acid	*L. casei*	Broadbent et al., 2010	19
Decrease in unsaturated/saturated fatty acid ratio	Acid	*L. delbrueckii*	Streit et al., 2008	20
Decrease in unsaturated/saturated fatty acid ratio	Osmotic	*L. casei*	Machado et al., 2004	21
Increase in unsaturated/saturated fatty acid ratio	Bile salts	*L. reuteri*	Taranto et al., 2003	22
Increase in unsaturated/saturated fatty acid ratio	Cold	*L. acidophilus*	Wang et al., 2005	23
Increase in unsaturated/saturated fatty acid ratio	Heat	*L. helveticus*	Lanciotti et al., 2001	24
Increase in unsaturated/saturated fatty acid ratio	Oxidatif	*L. helveticus*	Lanciotti et al., 2001	24
Decrease in the number of cycloporpane fatty acids	Acid	*L. delbrueckii*	Streit et al., 2008	20
Decrease in the number of cycloporpane fatty acids	Acid	*L. bulgaricus*	Li et al., 2009	23
Increase in the number of cycloporpane fatty acids	Acid	*L. casei*	Broadbent et al., 2010	19
Increase in the number of cycloporpane fatty acid	Acid	*L. acidophilus*	Wang et al., 2005	23
Increase in the number of cycloporpane fatty acid	Heat	*L. bulgaricus*	Li et al., 2009	25
Increase in S-layer production	Acid	*L. acidophilus*	Khaleghi and Kasra, 2012	26
Increase in S-layer production	Bile salts	*L. acidophilus*	Khaleghi et al., 2010; Grosu-Tudor et al., 2016	27, 28
Increase in S-layer production	Heat	*L. acidophilus*	Khaleghi and Kasra, 2012; Grosu-Tudor et al., 2016	27, 26
Increase in S-layer production	Osmotic	*L. acidophilus*	Palomino et al., 2013, 2016; Grosu-Tudor et al., 2016	27, 29, 31
Thinning of the cell wall	Osmotic	*L. casei*	Piuri et al., 2005	30
Increase in the surface hydrophobicity	Osmotic	*L. casei*	Machado et al., 2004	21
Reduction of lipotecoïc acid	Osmotic	*L. casei*	Palomino et al., 2013	29
Increase in the negative charge of the cell wall	Osmotic	*L. casei*	Palomino et al., 2013	29
Reduction of the lipotecoïc chain	Osmotic	*L. casei*	Palomino et al., 2013	29
Increase in SlpA/SlpX ratio	Osmotic	*L. acidophilus*	Palomino et al., 2016	31
Increase in the density and the thickness of the cell wall	Addition of transglutaminase	*L. lactis*	Li et al., 2015	32
ClpB overproduction	Acid	*L. plantarum*	Bove et al., 2013	33
ClpB overproduction	Bile salt	*P. freudenreichii*	Leverrier et al., 2004	34
ClpB overporudction	Heat	*B. breve*	De Dea Lindner et al., 2007	35
ClpB overproduction	Heat	*P. freudenreichii*	Leverrier et al., 2004	34
ClpB overporudction	Osmotic	*B. breve*	De Dea Lindner et al., 2007	35
ClpB overproduction	Osmotic	*P. freudenreichii*	Leverrier et al., 2004; Huang et al., 2016b	6, 34
ClpC overproduction	Acid	*P. freudenreichii*	Leverrier et al., 2004	34
ClpE overproduction	Acid	*L. plantarum*	Bove et al., 2013	33
ClpP overproduction	Acid	*L. plantarum*	Bove et al., 2013	33
CspA overproduction	Cold	*P. freudenreichii*	Dalmasso et al., 2012a	9
CspB overproduction	Cold	*P. freudenreichii*	Dalmasso et al., 2012a	9
CspC overproduction	Cold	*L. plantarum*	Derzelle et al., 2000	36
CspL overproduction	Cold	*L. plantarum*	Derzelle et al., 2000	36
CspP overproduction	Cold	*L. plantarum*	Derzelle et al., 2000	36
DnaJ1 overproduction	acid	*B. longum*	Jin et al., 2015	37
DnaJ1 overproduction	Heat	*B. breve*	De Dea Lindner et al., 2007	35
DnaJ1 overproduction	Osmotic	*B. breve*	De Dea Lindner et al., 2007	35
DnaK overproduction	Acid	*L. delbrueckii*	Lim et al., 2001; Gouesbet et al., 2002	38, 39
DnaK overproduction	Bile salt	*P. freudenreichii*	Leverrier et al., 2004	34
DnaK overproduction	Bile salt	*P. freudenreichii*	Savijoki et al., 2005	40
Dnak overproduction	Heat	*B. breve*	De Dea Lindner et al., 2007	35
DnaK overproduction	Heat	*P. freudenreichii*	Savijoki et al., 2005	40
DnaK overproduction	Heat	*L. rhamnosus*	Prasad et al., 2003	41
Dnak overproduction	Osmotic	*B. breve*	De Dea Lindner et al., 2007	35
DnaK overproduction	Osmotic	*P. freudenreichii*	Leverrier et al., 2004	34
DnaK overproduction	Heat	*P. freudenreichii*	Leverrier et al., 2004	34
GroEL overproduction	Acid	*L. delbrueckii*	Lim et al., 2001; Gouesbet et al., 2002	38, 39
GroEL overproduction	Acid	*P. freudenreichii*	Jan et al., 2001; Leverrier et al., 2004	42, 34
GroEL overproduction	Bile salt	*P. freudenreichii*	Savijoki et al., 2005	40
GroEL overproduction	Heat	*B.breve*	Ventura et al., 2004	43
GroEL overproduction	Heat	*L. rhamnosus*	Prasad et al., 2003	41
GroEL overproduction	Heat	*P. freudenreichii*	Savijoki et al., 2005	40
GroES overproduction	Acid	*L. delbrueckii*	Lim et al., 2001; Silva et al., 2005	39, 44
GroES overproduction	Acid	*P. freudenreichii*	Jan et al., 2001	42
GroES overproduction	Heat	*B. breve*	Ventura et al., 2004	43
GroESL overproduction	Heat	*L. johnsonii*	Walker et al., 1999	45
grpE overproduction	Heat	*B. breve*	De Dea Lindner et al., 2007	35
grpE overproduction	Osmotic	*B. breve*	De Dea Lindner et al., 2007	35
HtrA overproduction	Bile salt	*P. freudenreichii*	Savijoki et al., 2005	40
HtrA overproduction	Heat	*P. freudenreichii*	Savijoki et al., 2005	40
SodA overproduction	Bile salt	*P. freudenreichii*	Leverrier et al., 2004	34
SodA overproduction	Heat	*P. freudenreichii*	Leverrier et al., 2004	34
SodA overproduction	Osmotic	*P. freudenreichii*	Leverrier et al., 2004	34


*^+^ indicates an improvement of survival. ^-^ indicates a decrease of survival. ^0^ indicates no effect on survival. ^∗^ indicates a cited reference. ^*a*^*Lactobacillus*. ^*b*^*Propionibacterium*. ^*c*^*Lactococcus*. ^*d*^ Bifidobacterium*.

### Accumulation of Compatible Solute and Energy Storage

A compatible solute is a small organic molecule that is polar, highly soluble in water, and that has a neutral isoelectric point. It behaves like an osmolyte, allowing a live cell to adapt to an osmotic stress (Csonka, 1989). During osmotic stress, bacteria accumulate compatible solutes (Csonka, 1989), either transported from the external medium, or synthesized *de novo*, to restore turgescent pressure and enable cell growth and division (Csonka and Hanson, 1991). Compatible solutes are unable to rapidly cross bacterial membranes without the involvement of a transport system and, for the most part, do not carry an electrical charge at a neutral pH. Uncharged molecules can be accumulated at a high concentration without disturbing the metabolism (Csonka, 1989). There are a limited number of molecules considered to be compatible solutes, and they can be divided into two categories: the first one corresponds to sugars and polyols and the second is composed of alpha and beta amino acids and their derivatives (Roesser and Müller, 2001). Compatible solutes preserve the conformation of proteins submitted to osmotic constraint. Generally, compatible solutes are excluded from the immediate vicinity of the proteins by an unfavorable interaction between the protein surface and the compatible solutes. This mechanism is known as “preferential excluding” (Roesser and Müller, 2001).

#### Accumulation of Sugar and Polyol

##### Accumulation of trehalose

**FIGURE 2:**
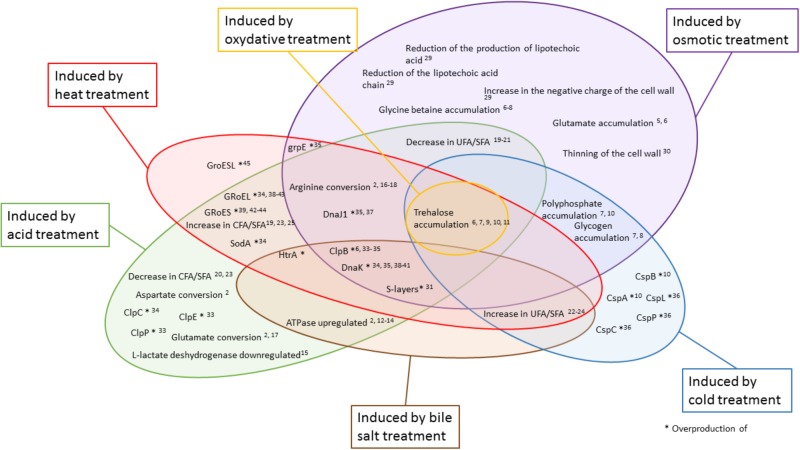
Different treatments modulate the key actors of adaptive mechanisms. Colored areas represent the different treatments studied. In yellow: oxidative; in red: heat; in green: acid; in brown: bile salts; in blue: cold; and in purple: osmotic treatment. The key actors of adaptive mechanisms indicated inside a bubble are modulated by the corresponding treatment. The numbers indicate corresponding references in the tables.

The versatile role of trehalose accumulation in stresses adaptation is schematized in Figure 2. Indeed, trehalose is a stable molecule (Giulio et al., 2005) which prevents protein aggregation, facilitates refolding, and protects cells and cellular proteins from damages caused by oxygen (Cardoso et al., 2004). It is also suggested that trehalose plays a role in the stabilization of cell membranes (Cardoso et al., 2007). Trehalose can act as an intracellular carbon stock and is consumed after exhaustion of the external carbon source (Cardoso et al., 2004). High sugar concentration in the culture medium promotes trehalose accumulation. Osmotic stress triggers accumulation of trehalose in *P. freudenreichii* and *L. casei* (Cardoso et al., 2004; Huang et al., 2016b). The accumulation of compatible solutes is facilitated in a rich medium as opposed to a chemically defined mediaTrehalose accumulation by *P. freudenreichii* may also be induced by other stresses such as cold (Dalmasso et al., 2012b), oxidative, and acid stresses (Cardoso et al., 2004, 2007). Moreover, trehalose can decrease the loss of viability during storage after freeze-drying (Giulio et al., 2005).

##### Accumulation of glycerol

Huang et al. (2016b) observed that Glycerol 3–phosphate dehydrogenase was overexpressed by *P. freudenreichii* in hyperconcentrated sweet whey medium. During osmotic stress, glycerol modification on oligosaccharides is increased. This phenomenon induces a possible increase of hydroxyl groups available number in saccharide molecules and replaces water molecules for cellular interaction, during osmotic stress, for example (Prasad et al., 2003).

#### Accumulation of Amino Acids

In osmotic adaptation, lactobacilli regulate the intracellular concentration of amino acids like proline and glutamate (Papadimitriou et al., 2016). Glutamate is a key metabolite which plays a role in various cell stress responses (Feehily and Karatzas, 2013). This amino acid is crucial for the primary response to hyper-osmotic shock (Glaasker et al., 1996). The first response to a high osmolarity is the accumulation of K^+^ and its counterion, glutamate. Glutamate has a minor influence on the acid tolerance capacity of *Propionibacterium acidipropionici* (Guan et al., 2013). The addition of glutamate in the growth medium enhanced glutamate accumulation by *L. sakei*, even in the absence of stressing conditions (Ferreira et al., 2005).

An increase in lysine production during stress has been observed in lactobacilli (Papadimitriou et al., 2016). In *Propionibacterium acidopropionicii*, a similar phenomenon was observed for other amino acids such as arginine and aspartate, which were accumulated during acid adaptation (Guan et al., 2013). GABA (decarboxylation of glutamate to γ-aminobutyrate) is involved in acid tolerance (Bron et al., 2006; Guan et al., 2013). The lysine degradation pathway is activated under acidic conditions in *L. plantarum* (Heunis et al., 2014).

Glycine betaine is known to protect different bacterial species against high osmolarity and is considered to be the most effective osmoprotectant (Roesser and Müller, 2001). During osmotic stress, *P. freudenreichii* accumulation of glycine betaine occurs via the OpuABC (or Bus ABC) transporter, which is osmotically induced (Figure 1) (Huang et al., 2016b). The glycine betaine transporter OpuABC of *Lactococcus lactis* was also well described by Romeo et al. (2003), as well the QacT transporter in *L. plantarum* by Glaasker et al. (1998).

#### Accumulation of Energy Storage Compounds

##### Accumulation of phosphates

Polyphosphates are not only used for energy storage but they are also accumulated by *P. freudenreichii* during cold (Dalmasso et al., 2012a) and osmotic stresses (Huang et al., 2016b). For lactobacilli, the accumulation of polyphosphates is dependent on high phosphate concentration in the growth medium (Alcantara et al., 2014). Polyphosphates act as chaperones in other bacteria and interact with misfolded proteins in oxidative stress conditions (Gray and Jakob, 2015). Polyphosphate accumulation is recognized as a key factor of stress tolerance in *L. casei* (Huang et al., 2018).

The polyphosphate kinase, responsible for polyphosphate (Ppk) synthesis, catalyzes the ATP-dependent formation of a phosphoanhydride bond between a polyphosphate chain and orthophosphate. Some lactobacilli have more than one *ppk* gene involved in polyphosphate synthesis. The number of *ppk* genes was shown to be correlated with the accumulation of elevated phosphate concentrations (Alcantara et al., 2014).

##### Accumulation of glycogen

Low temperatures induce glycogen accumulation in *P. freudenreichii* (Dalmasso et al., 2012b). This bacterium also accumulates glycogen in hyper-concentrated sweet whey medium, which has a high osmotic pressure and provides an abundance of carbon substrate (Huang et al., 2016b). Glycogen accumulation depends on the type of sugar substrate present in the culture medium. Raffinose and trehalose activate the accumulation of glycogen, whereas glucose represses it in lactobacilli (Goh and Klaenhammer, 2014).

### Regulation of Energy Production

Intracellular pH (pHi) homeostasis is a prerequisite to normal growth or to survival during stress (Guan et al., 2013). Bacteria triggered different mechanisms to regulate their pHi, such as the upregulation of ATPase activity and the conversion of different substrates.

#### Regulation of ATPase Activity

Lactobacilli acid tolerance is attributed to the presence of a constant gradient between extracellular and pHi. The ATPase protein is a known mechanism used for protection against acid stress (Figure 1). This protein generates a proton driving force via proton expulsion (Corcoran et al., 2005; Guan et al., 2013). The mechanism is similar for bifidobacteria during acid (Sanchez et al., 2007) and bile salts stresses (Sanchez et al., 2006). The regulation of ATPase activity occurs at the transcriptional level (Broadbent et al., 2010). There is a correlation between APTase activity and acid tolerance (Guan et al., 2013): the higher the ATPase activity is, the higher the acid tolerance will be. However, some lactobacilli species (like *L. casei*) do not use ATPase as a tolerance response to acid stress, instead, they keep the pHi low and reduce the energy demand for proton translocation, in addition to preventing intracellular accumulation of organic acid (Broadbent et al., 2010).

#### Regulation of Substrate Conversion

During an acid stress, lactobacilli decrease the production of lactate (Papadimitriou et al., 2016). In cold stress conditions, propionibacteria decrease the production of propionate and acetate from lactate substrate via a redirection of pyruvate from the Wood-Werkman pathway to other metabolic pathways (Dalmasso et al., 2012a). To limit the decrease of the pHi, *P. acidopropionici* and lactobacilli increase the activity of the arginine deaminase (ADI) system by a factor of three to five. This system allows the degradation of arginine, producing ATP, NH_4_^+^, and CO_2_ (Rollan et al., 2003; Guan et al., 2013; Teixeira et al., 2014). The production of NH_4_^+^ and CO_2_ allows pH homeostasis (Guan et al., 2013), and the ATP produced leads to an exclusion of protons by the APTase. Guan et al. (2013) showed that the conversion of aspartic acid into alanine makes it possible for the latter to contribute to the ADI system. In addition, ADI induction can also occur during osmotic and heat stress in *L. fermentum* (Vrancken et al., 2009). During acid treatment, *Lactobacillus reuteri* overexpresses glutamine deaminase and glutamate decarboxylase (Su et al., 2011; Teixeira et al., 2014) (Figure 1). These enzymes also generate NH_4_^+^ and CO_2_ and are involved in pH_i_ homeostasis.

At a low pH, enzymes involved in catabolism and energy production are overproduced (Sanchez et al., 2007). This includes enzymes involved in the consumption of complex carbohydrates that contribute to the bifid shunt (Sanchez et al., 2007). Carbon utilization is more efficient and produces more ATP, which could contribute to the proton exclusion via ATPase (Sanchez et al., 2007). The addition of glucose in the growth medium can improve acid tolerance by providing the ATP pool required for proton extrusion by ATPase (Corcoran et al., 2005). NAD is used in glycolysis so that the NAD/NADH ratio has to be optimized for high ATP production (Guan et al., 2013).

### Impact on the Bacterial Envelope

Lactobacilli, propionibacteria, and bifidobacteria encountered membranes damages to during various stresses (Papadimitriou et al., 2016). Indeed, the cytoplasmic membrane acts as a barrier for most solutes. The cell membrane also plays a role in other stresses like acid, cold, heat, and bile salts (Table 1). The cell envelope plays also a key role in the regulation of osmotic stress; and maintains cell shape and counteracts the high intracellular osmotic pressure (Papadimitriou et al., 2016). To restore membrane and cell wall integrity, different adaptation mechanisms are adopted by bacteria.

#### Regulation of Membrane Fluidity

The membrane has such an important role that the relationship between membrane fluidity and stress tolerance has been used to predict the outcome of cell tolerance to stress (Muller et al., 2011). Indeed, stability and permeability of membranes are both key parameters of adaptation and tolerance toward various stresses (Machado et al., 2004). Modulation of membranes composition as adaptation mechanism, which can occur under stressing conditions, tends to counteract variations of fluidity in order to maintain the structure of the bilayer (Figure 1).

Changes in membrane lipids composition are strain-dependent. As presented in Figure 2, the cyclic/saturated fatty acid ratio can be increased or decreased during osmotic stress. During osmotic adaptation, an increase in the amount of cyclopropane fatty acids is observed in *L. lactis*, while the unsaturated/saturated ratio in membrane fatty acids remains unchanged (Guillot et al., 2000). However, for other bacteria, a decrease in the unsaturated/saturated lipid ratio in the membrane composition can be observed during osmotic adaptation (Machado et al., 2004). *L. casei* can do both during acid stress, increasing the number of cyclopropane fatty acids and decreasing the unsaturated/saturated ratio. In acidic conditions, bacteria have to counteract proton influx by increasing the rigidity and compactness of the cytoplasmic membrane (Broadbent et al., 2010). The unsaturated/saturated ratio and the cyclic/saturated ratio decrease in *L. delbreuckii* subsp. *bulgaricus* in membrane under acidic conditions, thus leading to a decrease in membrane fluidity (Streit et al., 2008). During heat stress, *L. helveticus* decreases its membrane fluidity by increasing the number of unsaturated fatty acids (Lanciotti et al., 2001). *P. freudenreichii*, which contains a majority of odd-numbered membrane unsaturated fatty acids, changes its fatty acid composition under cold stress conditions were observed. It reduces the amount of iso-fatty acids in favor of anteiso-fatty acids (Dalmasso et al., 2012a). As an example, in propionibacteria, branched-chain fatty acids are synthesized due to the activity of branched alpha-keto acid dehydrogenase under cold stress during cheese storage (Dalmasso et al., 2012a). *P. freudenreichii* uses different enzymes to synthesize branched-chain fatty acids, which result from the catabolism of branched amino acids (Gagnaire et al., 2015), maintaining the fluidity of the membrane in order to counteract the cold stress.

The role of cyclic fatty acids (poorly understood) includes the modulation of the fluidity in order to increase the tolerance to different stresses (Machado et al., 2004). Cyclic fatty acid concentration increases during the stationary phase (Muller et al., 2011). The number of double bonds in unsaturated fatty acids is important: linoleic and linolenic acids have two and three double bonds, respectively, causing more steric hindrances than oleic acid (one double bond). Several double bonds could result in the loss of membrane integrity and cell death (Muller et al., 2011). Indeed, *Lactobacillus johnsonii* NCC533 supplemented with unsaturated fatty acid shows a higher sensitivity to heat and acid stress.

Fatty acid biosynthesis, or neosynthesis, requires a great deal of energy, but bacteria have the possibility of modifying existing fatty acids. For example *L. casei* ATCC 334 possesses an enzyme that can add a methylene residue across the cis double bond of C16:1n(9), C18:1n(9), or C18:1n(11) unsaturated fatty acids to form a cyclopropane derivative, thus allowing bacterial adaptation with a minimal energy requirement (Broadbent et al., 2010).

Modulation of the membrane bilayer fatty acid composition seems to be stress-dependent as well as strain-dependent (Figure 2). Different bacteria reach the same goal, environmental adaptation, in different ways.

#### Regulation of the Cell Wall

Peptidoglycan is a key element for the stability of bacteria. It is composed of glycan chains of repeating *N*-acetyl-glucosamine and *N*-acetyl-muramic acid residues, and cross-linked by peptide side chains (Piuri et al., 2005). During osmotic treatment, bacteria are affected by an enlargement of the cell (Piuri et al., 2005). During enlargement, the cell wall loses a layer. Two layers can be observed in high salt conditions, whereas three layers are observed under normal conditions. The cell wall is thus thinned (Figure 1). Under salt stress adaptation, the structure is irregular and seems to be detached from the cytoplasmic membrane. This phenomenon can be attributed to plasmolysis. The presence of protein and teichoic acid in controlled conditions may be responsible for the presence of a third layer (Piuri et al., 2005).

Cells grown in high osmolarity increase the hydrophobicity of their surface, revealed by a higher adherence to the organic solvent. In Gram-positive bacteria, lipoteichoic acids and proteins are the most important cell wall components responsible for surface hydrophobicity. This high hydrophobicity helps bacteria to tolerate the osmotic stress (Machado et al., 2004). *L. casei* grown in high salt conditions limits its production of lipoteichoic acids. In addition, lipoteichoic acids exhibit a lower mean chain length and a lower D-alanine substitution during osmotic treatment. D-alanine substitution increases negative charges in the cell wall and contributes to bacteria tolerance by helping to evacuate toxic Na^+^ from the cell wall (Palomino et al., 2013).

#### Regulation of the Different S-Layers

S-layer proteins are the outer layer component and have several different functions; they maintain cell shape, provide a protective coating, and adhere to the host cell. Lactobacilli overexpress S-layer proteins under stress conditions like bile salts, acid, heat, and salt stresses (Khaleghi et al., 2010; Khaleghi and Kasra, 2012; Grosu-Tudor et al., 2016). Moreover, they are involved in osmoadaptation (Palomino et al., 2016). During osmotic treatment, there is an overproduction of the surface layer proteins A (SlpA) and X (SlpX), and the SlpA/SlpX ratio is modified in *L. acidophilus* (Palomino et al., 2016). S-layer proteins may have a role as a protective sheath and protect cells against mechanical and osmotic insults. Bacteria may increase the S-layer gene expression in order to maintain the integrity of the cell envelope structure (Figure 1), mainly because of the decrease of the cell wall thickness (Palomino et al., 2016).

#### Regulation of Exopolysaccharide

Exopolysaccharides include various forms of polysaccharides and are located outside the microbial cell wall. They consist of repeating units of homo- or heteropolysaccharides (Caggianiello et al., 2016). The exopolysaccharides can be strongly or weakly bound to the cell surface and protect bacteria against high temperature, acid, bile salts, and osmotic stress (Alp and Aslim, 2010; Stack et al., 2010; Caggianiello et al., 2016). Exopolysaccharide production is improved under acidic conditions (Torino et al., 2001) and enhances thus the bacterial tolerance response.

### Overexpression of Molecular Chaperones and Stress-Responsive Proteases

The synthesis of chaperones and proteases is quickly induced under various stressing conditions (Figure 2) to decrease the deleterious impact of the aggregation of denatured proteins and to refold misfolded ones. Proteases act like the last line of defense when damage is irreversible and leads to amino acids recycling. Proteolysis of cellular proteins, which is a regulated process, can greatly contribute to homeostasis by degrading proteins whose functions are no longer required after modification of environmental parameters (Papadimitriou et al., 2016). DnaK (Hsp70) is one of the well-conserved bacterial chaperones that can refold misfolded proteins (Papadimitriou et al., 2016).

One of the ways to counteract stress is the regulation of membrane fluidity. This rather complex regulation involves GroEL and HSPs universal protein chaperones (Papadimitriou et al., 2016). Indeed, small heat shock proteins regulate membrane lipid polymorphism (Tsvetkova et al., 2002). Accordingly, inactivation of small heat shock proteins affects membrane fluidity in *L. plantarum* (Capozzi et al., 2011). GroEL and GroES (Hsp60) proteins are overproduced under various stress conditions (Papadimitriou et al., 2016) (Figure 2). It has been shown that *Lactobacillus paracasei* overproduces GroESL during heat stress (Santivarangkna et al., 2007). GroEL and GroES work in tandem to ensure the correct folding of proteins in an ATP-regulated manner (Mills et al., 2011) (Figure 2).

De Dea Lindner et al. (2007) showed that there are two types of stress protein regulation. In the first type, GroEL, GroES, and ClpC are rapidly induced to a high level after moderate heat stress (+5°C) but are not induced by severe heat treatment (+13°C). In the second type, DnaK, GrpE, DnaJ1, and ClpB are strongly induced in a high heat treatment but not in moderate one (De Dea Lindner et al., 2007). Heat shock proteins are highly conserved but it is not the case for their expression regulation mechanisms. The heat shock response is controlled by a combination system like transcriptional repression and activation (De Dea Lindner et al., 2007).

*Bifidobacterium breve* overexpresses DnaK, DnaJ, and ClpB in high salt conditions and during high heat treatment, which probably means that there is an overlapping regulatory network that controls both osmotic and severe heat stress (De Dea Lindner et al., 2007). This is consistent with observed cross-protections between heat and salt treatments. These proteins are also overproduced during bile and acid stresses (Leverrier et al., 2004; Bove et al., 2013; Jin et al., 2015).

In addition, *P. freudenreichii* overproduces HtrA, DnaK, and GroEL during bile salts stress, and HtrA is known to have protease and chaperone activities (Leverrier et al., 2003, 2004). This suggests that bile and salt adaptation are closely related (Savijoki et al., 2005).

The Clp protease family constitutes a major system for general protein turnover in lactobacilli. These proteins work with ATPase to degrade proteins (Papadimitriou et al., 2016). Chaperones and Clp proteins are stress-induced, with induction occurring at the transcription level, and can be regulated by the transcriptional repressors CtsR and HrcA (Papadimitriou et al., 2016).

Cold shock proteins (Csp) are produced in cold conditions (Figure 2) and are involved in mRNA stabilization (Mills et al., 2011). These proteins are essential for growth in cold temperatures (Dalmasso et al., 2012a). The production of three Csps, CspL, CspP, and CspC, in cold conditions resulted in an increased of bacterial tolerance toward temperature downshift (Mills et al., 2011). In cold and other stress conditions, *P. feudenreichii* upregulates genes coding for two Csp (Dalmasso et al., 2012a). Bifidobacteria have less different chaperones and proteases than other bacteria (De Dea Lindner et al., 2007), in accordance with their great sensitivity to stress.

Chaperones can also behave as moonlight proteins. Moonlighting DnaK and GroEL, for example, have adhesive functions. The overexpression of these proteins during bile or acid stresses can help the bacteria to adhere to intestinal cells and to persist within the digestive tract (Bergonzelli et al., 2006; Candela et al., 2010).

The whole studies reveal that some adaptive mechanisms are common to different treatments. They may be induced by several treatments and explain the observed cross-protections (Figure 2). Osmotic and acid treatments induce large changes in bacterial cells, and only a few adaptation mechanisms are common to both treatments. Oxidative treatment has been less studied than the other treatments.

## Stress Adaptation Improves Bacterial Tolerance to Technological and Digestive Stress

**FIGURE 3:**
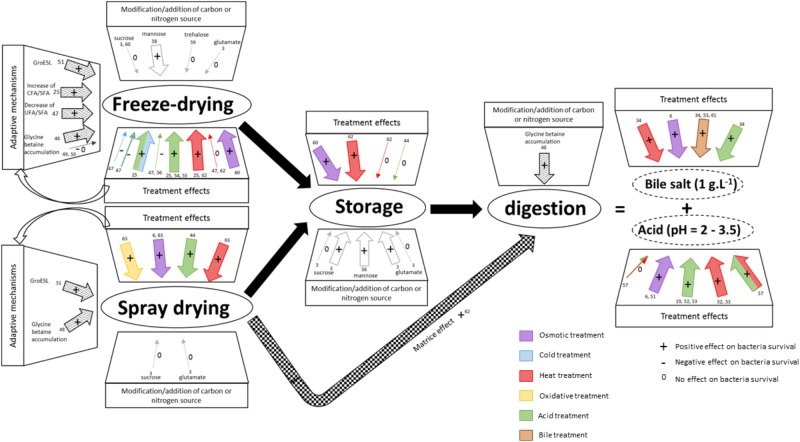
Stressing pretreatments and modifications of the growth medium modulate survival during technological and digestive stresses. Technological and digestive stresses are represented in the figure. Digestion triggers two main stresses: bile salts and acid stress. For each stress, the impact of stressing pretreatments and of modifications of the growth medium on bacteria survival is indicated (+: positive; -: negative; and 0: no effect). Strain-dependent modulations are represented by arrows (purple: osmotic; blue: cold; red: heat; yellow: oxidative; green: acid; brown: bile salts treatment). Large arrows indicate a positive effect and thin arrows indicate either no effect or a negative effect. The numbers indicate corresponding references in the tables.

Understanding and mastering bacterial adaptation is crucial for strains selection and of pretreatments to improve viability during technological stresses, storage, and digestion (Figure 3). Bacterial survival during technological and digestive stresses is strain-dependent. Some bacteria are naturally more resistant and have the ability to adapt to various stresses. Some growth medium modifications and some physicochemical treatments can improve bacterial survival during technological stresses, thanks to adaptation (Tables 2, 3 and Figure 3), but only a few studies have focused on which adaptive mechanisms are responsible for enhanced survival.

**Table 2:** Adaptive mechanisms reported to modulate the survival of propionibacteria, bifidobacteria, and lactobacilli under technological and digestive stresses.

Cell modification	Effect	Technological stress or digestion	Bacteria	References	Corresponding number in the figures
Conversion of glutamate to GABA	+	Acid stress (pH = 2.5)	*L.*^a^ *reuteri*	Su et al., 2011	46^∗^
Decrease in unsaturated fatty acid/saturated fatty acid ratio	+	Freeze-drying	*L. coryniformis*	Schoug et al., 2008	47
Glycine betaine accumulation	+	Digestion	*B.*^b^ *breve*	Sheehan et al., 2006	48
Glycine betaine accumulation	0	Freeze-drying	*B. animalis*	Saarela et al., 2005	49
Glycine betaine accumulation	-	Freeze-drying	*L. coryniformis*	Bergenholtz et al., 2012	50
Glycine betaine accumulation	+	Freeze-drying	*L. salivarius*	Sheehan et al., 2006	48
Glycine betaine accumulation	+	Spray-drying	*L. salivarius*	Sheehan et al., 2006	48
GroESL overproduction	+	Freeze-drying	*L. paracasei*	Corcoran et al., 2006	51
GroESL overproduction	+	Spray-drying	*L. paracasei*	Corcoran et al., 2006	51
Increase in cyclopropane fatty acid number	+	Freeze-drying	*L. bulgaricus*	Li et al., 2009	25


*^+^ indicates an improvement of survival. ^-^ indicates a decrease of survival. ^*0*^ indicates no effect on survival. ^∗^ indicates a cited reference. ^*a*^*Lactobacillus*. ^*b*^ Bifidobacterium*.

**Table 3:** Treatments and modifications of the growth medium that modulate the survival of propionibacteria, bifidobacteria, and lactobacilli under technological and digestive stresses.

Adaptation	Effect	Technological stress or digestion	Bacteria	References	Corresponding number in the figures
Acid adaptation	+	Acid stress (pH = 2)	*L.*^a^*casei*	Broadbent et al., 2010	19^∗^
Acid adaptation	+	Acid stress (pH = 2)	*P.^b^ freudenreichii*	Jan et al., 2000, 2002	52, 53
Acid adaptation	-	Bile salt stress (1g.L-1)	*P. freudenreichii*	Leverrier et al., 2004	34
Acid adaptation	+	Freeze-drying	*L. bulgaricus*	Li et al., 2009	25
Acid adaptation	-	Freeze-drying	*L. coryniformis*	Schoug et al., 2008	47
Acid adaptation	+	Freeze-drying	*L. reuteri*	Palmfeldt and Hahn-Hägerdal, 2000; Koch et al., 2008	54, 55
Acid adaptation	-	Freeze-drying	*L. rhamnosus*	Ampatzoglou et al., 2010	56
Acid adaptation	+	Spray-drying	*L. delbrueckii*	Silva et al., 2005	44
Acid adaptation	0	Storage (SD)	*L. delbrueckii*	Silva et al., 2005	44
Cold + acid adaptation	-	Freeze-drying	*L. coryniformis*	Schoug et al., 2008	47
Cold + acid adaptation	+	Freeze-drying	*L. bulgaricus*	Li et al., 2009	25
Acid + heat adaptation	+	Acid stress (pH = 3,5 in Synthetic Gastric Fluid)	*B.*^c^ *lactis*	Maus and Ingham, 2003	57
Acid + heat adaptation	0	Acid stress (pH = 3,5 in Synthetic Gastric Fluid)	*B. longum*	Maus and Ingham, 2003	57
Addition of mannose	+	Freeze-drying	*L. delbrueckii*	Carvalho et al., 2008	58
Addition of mannose	+	Storage (FD)	*L. delbrueckii*	Carvalho et al., 2008	58
Addition of trehalose	0	Freeze-drying	*L. salivarius*	Zayed and Roos, 2004	59
Addition of glutamate	0	Freeze-drying	*L. sakei*	Ferreira et al., 2005	3
Addition of glutamate	0	Spray-drying	*L. sakei*	Ferreira et al., 2005	3
Addition of glutamate	0	Storage (FD)	*L. sakei*	Ferreira et al., 2005	3
Addition of glutamate	+	Storage (SD)	*L. sakei*	Ferreira et al., 2005	3
Addition of sucrose	0	Freeze-drying	*L. sakei*	Ferreira et al., 2005	3
Addition of sucrose	0	Freeze-drying	*L. bulgaricus*	Carvalho et al., 2003	60
Addition of sucrose	0	Spray-drying	*L. sakei*	Ferreira et al., 2005	3
Addition of sucrose	+	Storage (SD)	*L. sakei*	Ferreira et al., 2005	3
Addition of sucrose	0	Storage (FD)	*L. sakei*	Ferreira et al., 2005	3
Bile salt adaptation	+	Bile salt stress (1 g.L^-1^)	*P. freudenreichii*	Jan et al., 2002; Leverrier et al., 2003, 2004	53, 61, 34
Cold adaptation	-	Freeze-drying	*L. coryniformis*	Schoug et al., 2008	47
Heat adaptation	+	Acid stress (pH = 2)	*P. freudenreichii*	Jan et al., 2000, 2002	52, 53
Heat adaptation	+	Bile salt stress (1 g.L^-1^)	*P. freudenreichii*	Leverrier et al., 2004	34
Heat adaptation	0	Freeze-drying	*L. acidophilus*	Paéz et al., 2012	62
Heat adaptation	+	Freeze-drying	*L. bulgaricus*	Li et al., 2009	25
Heat adaptation	+	Freeze-drying	*L. casei*	Paéz et al., 2012	62
Heat adaptation	0	Freeze-drying	*L. coryniformis*	Schoug et al., 2008	47
Heat adaptation	+	Freeze-drying	*L. plantarum*	Paéz et al., 2012	62
Heat adaptation	+	Spray-drying	*L. paracasei*	Desmond et al., 2001	63
Heat adaptation	0	Storage (FD)	*L. acidophilus*	Paéz et al., 2012	61
Heat adaptation	0	Storage (FD)	*L. casei*	Paéz et al., 2012	61
Heat adaptation	+	Storage (FD)	*L. plantarum*	Paéz et al., 2012	61
Osmoadaptation	+	Acid stress (pH = 2)	*P. freudenreichii*	Jan et al., 2000; Huang et al., 2016b	6, 51
Osmoadaptation	+	Bile salt stress (1 g.L^-1^)	*P. freudenreichii*	Huang et al., 2016b	6
Osmoadaptation	+	Freeze-drying	*L. bulgaricus*	Carvalho et al., 2003	60
Osmoadaptation	+	Spray-drying	*L. paracasei*	Desmond et al., 2001	63
Osmoadaptation	+	Spray-drying	*P. freudenreichii*	Huang et al., 2016b	6
Osmoadaptation	+	Storage (FD)	*L. bulgaricus*	Carvalho et al., 2003	60
Oxidative adaptation	+	Spray-drying	*L. paracasei*	Desmond et al., 2001	63
Spray-dried	+	Digestion	*L. acidophilus*	Paéz et al., 2012	61
Spray-dried	+	Digestion	*L. casei*	Paéz et al., 2012	61


*^*+*^ indicates an improvement of survival. ^*-*^ indicates a decrease of survival. ^*0*^ indicates no effect on survival. ^∗^ indicates a cited reference. ^*a*^*Lactobacillus*. ^*b*^*Propionibacterium*. ^*c*^ Bifidobacterium.*

### Enhanced Tolerance Toward Drying Process and Storage

#### Freeze-Drying Process

Bacteria survival during freeze-drying has been well studied. Many studies presenting the best way to increase survival are available. However, only few studies have focused on the role of bacterial adaptive mechanisms on bacterial survival during this process.

The accumulation of glycine betaine leads to an enhanced survival during freeze-drying for *Lactobacillus salivarus* (Sheehan et al., 2006). Unfortunately, this mechanism is strain-dependent, as shown in Figure 3. Glycine betaine accumulation can be triggered by osmotic stress (Table 1; Glaasker et al., 1996; Romeo et al., 2003; Huang et al., 2016b). Indeed, as listed in Table 3, the osmoadaptation enhanced *Lacotbacillus bulgaricus* survival during freeze-drying (Carvalho et al., 2003). Osmotic constraint induces bacterial adaptation, and glycine betaine accumulation can be responsible for the better survival of *L. bulgaricus* and of other species. Figure 3 shows that acid, heat, and osmotic treatments can improve bacterial survival during freeze-drying in strain-dependent way. Osmotic stress can be also induced by the addition of either salt or sugar. The advantage of a sugar like mannose is that it can increase bacterial survival during freeze-drying (Carvalho et al., 2008). In fact, a growth medium with a high sugar concentration leads to the accumulation of trehalose (Cardoso et al., 2004). In addition, sugars have a positive impact on bacterial survival when they are added at a high concentration to the drying medium (Giulio et al., 2005).

A strain of *L. paracasei* which overexpresses GroESL presents an improved survival rate after extreme stresses challenges than other *L. paracasei* strains (Mills et al., 2011). GroESL is a chaperone protein which can be induced during heat treatment (Walker et al., 1999). The overproduction of GroESL or of other general stress proteins is induced by various treatments (Schoug et al., 2008) (Table 1), and their synthesis probably has a considerable impact on bacterial survival during freeze-drying.

Modulation of membrane lipids composition may contribute to enhancing bacterial survival during freeze-drying. In fact, lipid membrane modifications depend on the strain-treatment couple, like other adaptive mechanisms. In the literature, two lipid modifications have been reported to increase bacterial survival during freeze-drying. It includes the increase of the cyclic fatty/saturated fatty acid ratio and the decrease of the unsaturated/saturated membrane fatty acid ratio, these ratios being key features of stress adaptation (see section “Regulation of Membrane Fluidity”) (Table 2 and Figure 3).

Combinations of stress seem to be a promising way to induce multiple adaptation mechanisms, which unfortunately are strain-dependent. The combination of cold and acid treatment was tested, but this association decreased *Lactobacillus coryniformis* survival during freeze-drying (Schoug et al., 2008). However, Li et al. (2009) demonstrated that *L. bulgaricus* viability is enhanced during freeze-drying, when it is grown in cold and acid conditions (30°C; pH = 5) or when it is submitted to a mild cold treatment (30°C).

#### Spray-Drying Process

To enhance the latter, it is possible first to select strains with high intrinsic stress tolerance and the ability to adapt upon pretreatment, which is strain-dependent. Indeed, bifidobacteria species with high heat and moderate oxygen tolerance have a better survival rate after spray-drying (68–102%) compared to species with no intrinsic tolerance to oxygen and to heat stress (Santivarangkna et al., 2007). In addition, it is important to remember that grow phase harvesting may influence the bacterium viability during spray-drying. The stationary phase seems to be a favorable phase to harvest bacteria for drying (Peighambardoust et al., 2011).

The three treatments – osmotic, heat, and acid – can improve bacterial survival during spray-drying or freeze-drying (Figure 3). Although these two drying processes impose opposite stresses – cold and heat stress – the treatments that improve bacterial survival are indeed the same. It is possible to rank treatments according to their efficiency toward protection during spray-drying for *L. paracasei* NFBC 338. Decreasing efficiency is observed with heat > salt > hydrogen peroxide > and bile treatment (Desmond et al., 2001). Obviously, this ranking is strain-dependent and the addition of another treatment may make more effective bacterial adaptation possible.

The heat tolerance of bacteria is defined by the decimal reduction value (D_𝜃_ value) that represents the time needed to kill 90% of the bacteria at a given temperature 𝜃. Upon heat adaptation, the D_60_ value of *L. paracasei* can increase from 1.7 to 3.1 min (Desmond et al., 2001), in accordance with enhanced tolerance toward spray-drying. Survival of *L. salivarius* and *L. paracasei* is also enhanced by heat or oxidative treatments before spray-drying (Desmond et al., 2001). Moreover, during heat stress, GroESL can be overproduced and enhance bacterial survival during spray-drying (Walker et al., 1999; Corcoran et al., 2006). Silva et al. (2005) showed that acid-adapted *L. delbrueckii* achieves a better tolerance to heat and spray-drying due to the production of heat shock proteins.

In addition, the accumulation of glycine betaine improves lactic acid bacteria viability during spray-drying (Sheehan et al., 2006). High fermentable sugar concentrations in the growth medium permit the production of metabolites like mannitol. Non-fermentable sugars increase the osmotic pressure of the medium and induce osmoadaptation of bacteria, so that both types of sugars improve cell survival during spray-drying (Peighambardoust et al., 2011). Osmoadaptation increases the survival of several species during spray-drying (Desmond et al., 2001; Huang et al., 2016a,b).

Spray-drying is a stressful process that affects the bacterial membrane. Acid, osmotic, heat, and oxidative treatments lead to the modification of the membrane composition (Figure 2). Membrane fluidity should be optimized to increase bacteria survival. If the fluidity is too high or too low, bacterial survival may decrease.

Pretreatments prior to drying are thus promising tools to improve bacterial viability during technological processes, but must be adapted for each strain. It can be observed that bacterial adaptation has its own limits. As an example, the heat adaptation of *L. paracasei* would be useful in order to deal with high drying outlet temperatures (higher than 95°C) and, conversely, less useful for lower outlet temperatures (85–90°C) because the temperature would cause less damage in this case (Desmond et al., 2001). Moreover, the outlet temperature can be decreased while optimizing the drying parameters. It seems that the best pretreatment would be acid and osmotic stresses for spray-drying.

#### Storage

The challenge is not only to maintain the viability of bacteria during the drying process, but also during storage. Relative humidity and temperature are two key factors controlling the loss of viability upon storage. Since the oxidation of lipids increases with relative humidity (Golowczyc et al., 2011) and temperature, the stability is negatively correlated with these two factors. Cells have the best stability during storage with a relative humidity of 0%, even if the temperature is 30°C (Golowczyc et al., 2011).

To increase the stability of cells during storage, a protective agent can be added during growth. When monosodium glutamate or fructo-oligosaccharides (FOS) are added during the growth of *Lactobacillus kefir*, the cells have a higher stability after drying and during storage at 0–11% relative humidity (Golowczyc et al., 2011). Sugars like glucose, fructose, mannose, and sorbitol can provide protection (Peighambardoust et al., 2011). Protection through the addition of sucrose is strain-dependent (Golowczyc et al., 2011). Storage at relative humidity higher than 23% resulted in low stability, regardless of the protective agent, with a correspondingly low D_20_ value (Golowczyc et al., 2011). This highlights the importance of decreasing the relative humidity of the storage room.

Another way to prepare bacteria to storage conditions is cross-protection. Cells cultivated with a moderate stress can have better stability during storage. Only heat and osmotic treatments are reported to increase bacterial survival upon storage (Figure 3). Indeed, mild heat treatment enhances the stability of *L. paracasei* (Paéz et al., 2012) and of *Lactobacillus rhamnosus* (Prasad et al., 2003) during storage, these results being strain-dependent (Paéz et al., 2012).

Osmotic adaptation can also improve stability during storage for *L. rhamnosus* (Prasad et al., 2003). In powders, the absence of water may have deleterious effects on bacteria. Osmotic treatment during growth induces the accumulation of compatible solutes like glycine betaine, glycerol, and trehalose, and leads to changes in carbohydrate metabolism, resulting in the accumulation of glycerol linked to polysaccharides. These molecules interact with macromolecules instead of water. This protection increases the stability of *L. rhamnosus* during storage (Prasad et al., 2003). The modification of membrane composition during heat and osmotic treatments may reduce membrane damage, which occurs during storage.

*Lactobacillus delbrueckii* spp. *bulgaricus* grown under uncontrolled pH, thus experiencing acid stress, do not have a higher stability during storage (Silva et al., 2005).

### Enhance Tolerance Toward Digestive Stresses

During digestion, bile salts and acid stresses are two major stresses which affect probiotics survival and thus their beneficial effects. The effect of adaptation on bacterial survival during digestion is not well known (Figure 3).

Pre-adaptation of bifidobacteria to bile salts induces many metabolic changes. The first is the more efficient use of maltose by *Bifidobacterium animalis*. In addition, *Bifidobacterium longum* over-produces mucin-binding protein in acid pH conditions. This should facilitate its efficient targeting to the colon, which is its natural habitat. Moreover, *B. animalis* overproduces the membrane-bound ATPase that controls the pHi (Sánchez et al., 2013). Finally, adapted bifidobacteria are then able to consume raffinose and maltose, in addition to glucose (Sanchez et al., 2007). All these elements lead to a better survival within the gut, particularly because acid and bile salts adaptation prepare cells to use carbon sources, which cannot be used by the indigenous microbiota (Collado and Sanz, 2007; Sánchez et al., 2013).

Acid response is well documented for bifidobacteria. More than 20 bifidobacteria showed an increase of their tolerance to a simulated GIT after acid adaptation (Sánchez et al., 2013). Acid and heat adaptation can be used together at the same time to improve *Bifidobacterium lactis* survival to acid stress (Maus and Ingham, 2003).

Heat and osmotic treatments can be used to adapt bacteria to acid stress. In addition, *L. casei* can be acid-adapted with a treatment at pH 4.5 for 10 or 20 min, with an enhanced viability to acid challenge (Broadbent et al., 2010) and, consequently, to digestion. During bile salts adaptation, some probiotics express a range of bile salts hydrolases which lead a better bacterial viability (Papadimitriou et al., 2016). The spray-drying process can improve the tolerance to simulated gastrointestinal digestion as a result of encapsulation (Paéz et al., 2012) (Figure 3).

The probiotic delivery vehicle, used as drying matrix, has a high impact on viability during digestion. Several studies reported that spray-drying is the best method to maintain and improve the effect of probiotics. The protection of bacteria during digestion, both by encapsulation or by a food matrix, seems to be an important factor and has to be further studied in order to improve the effect of probiotics.

## Conclusion

Treatments used to adapt bacteria before technological and digestive challenges offer promising opportunities for improvement. Osmotic and heat treatments reportedly enhance bacteria survival during stressing challenges, via accumulation of compatible solutes and overexpression of key stress proteins, respectively. In the literature, these treatments are reported to have positive impacts on bacterial survival during freeze-drying, spray-drying, storage, and, to a lesser extent, during digestion. Acid treatment also seems promising because it can have a positive impact during drying, bile salts, and acid stress challenges. Treatments including bile salts have not been well studied, particularly in terms of their impact on bacterial survival during technological stresses. The combination of two or three of these stresses could be very interesting, especially the osmotic-acid combination. Indeed, it can be observed that these two treatments can induce a high number of adaptive mechanisms. Starter bacteria apart from two-in-one bacteria do not require an adaptation to digestive stress because they will grow in a food matrix. For starters, osmotic or heat treatment, or the combination thereof, seem to afford protection toward technological constraints. To trigger the adaptation of beneficial bacteria, the growth medium should be adapted, as carbon (saccharides) and nitrogen (amino acids) sources can be modulated. Growth conditions have to be chosen according to the subsequent probiotic/starter use and the strain since this review shows that most of the treatment effects are strain-dependent.

## Author Contributions

All authors contributed to the writing of the manuscript. GJ supervised the work.

## Conflict of Interest Statement

FG, PM, and PB were employed by the company Bioprox. The remaining authors declare that the research was conducted in the absence of any commercial or financial relationships that could be construed as a potential conflict of interest.
